# Ouabain Enhances Gap Junctional Intercellular Communication by Inducing Paracrine Secretion of Prostaglandin E2

**DOI:** 10.3390/ijms22126244

**Published:** 2021-06-10

**Authors:** Alejandro Ogazon del Toro, Lidia Jimenez, Mauricio Serrano Rubi, Marcelino Cereijido, Arturo Ponce

**Affiliations:** Department of Physiology, Biophysics and Neurosciences, CINVESTAV-IPN, Mexico City 07360, Mexico; alejandro.ogazon@cinvestav.mx (A.O.d.T.); lidia.jimenez@cinvestav.mx (L.J.); mauricio.serrano@cinvestav.mx (M.S.R.); cereijido@fisio.cinvestav.mx (M.C.)

**Keywords:** ouabain, gap junctions, dye transfer, prostaglandin E2

## Abstract

Ouabain is a cardiac glycoside that has been described as a hormone, with interesting effects on epithelial physiology. We have shown previously that ouabain induces gap junctional intercellular communication (GJIC) in wild, sensitive cells (MDCK-S), but not in cells that have become insensitive (MDCK-I) by modifying their Na^+^-K^+^-ATPase. We have also demonstrated that prostaglandin E2 (PGE2) is able to induce increased GJIC by a mechanism other than ouabain, that does not depend on Na^+^-K^+^-ATPase. In this work we show, by dye transfer assays, that when MDCK-S and MDCK-I are randomly mixed, to form monolayers, the latter stablish GJIC, because of stimulation by a compound released to the extracellular media, by MDCK-S cells, after treatment with ouabain, as evidenced by the fact that monolayers of only MDCK-I cells, treated with a conditioned medium (CM) that is obtained after incubation of MDCK-S monolayers with ouabain, significantly increase their GJIC. The further finding that either (1) pre-treatment with COX-2 inhibitors or (2) addition to CM of antagonists of EP2 receptor abolish CM’s ability to induce GJIC in MDCK-I monolayers indicate that PGE2 is the GJIC-inducing compound. Therefore, these results indicate that, in addition to direct stimulation, mediated by Na^+^-K^+^-ATPase, ouabain enhances GJIC indirectly through the paracrine production of PGE2.

## 1. Introduction

Gap junctions are molecular components of the cell membrane that allow communication between neighboring cells in animal tissues. They are channel-shaped structures, formed by proper docking of two hemichannels or connexons, provided by adjacent cells, which, when open, allow the exchange of ions and metabolites of low molecular weight between connecting cells. In turn, a connexon is an assembly of six proteins subunits called connexins forming a pore [1,2,3,4,5]. Ubiquitously expressed in animal tissues [6,7,8,9,10], gap junctions (GJ) are involved in coordinated actions of cells that make up a given tissue, including electrical communication, secretion, and control of growth and differentiation [11,12,13,14,15]. Given its participation in such a wide variety of physiological and pathological processes, it is important to understand how gap junctions work and how they are modulated.

Ouabain is a cardiac glycoside of remarkable interest due to its toxicological, pharmacological, and physiological properties. Initially extracted from plant sources, ouabain is a highly toxic compound, but at controlled concentration levels it has been used as a cardiotonic medicine, although it is no longer used for that purpose because it has a very narrow therapeutic range [16,17]. Paradoxically, ouabain has been shown to be expressed endogenously in some mammals, including humans, so it has been considered as a novel hormone [18,19,20] although, little is known, so far, about its physiological role.

The effects that ouabain cause depends on the dose at which it is used, however, it always does so through its interaction with Na^+^-K^+^-ATPase. At concentrations above 1 µM range ouabain acts by inhibiting the pumping action of Na^+^-K^+^-ATPase, while in concentrations in the nanomolar range it work as a hormone whereas Na^+^-K^+^-ATPase acts as a receptor that subsequently triggers signaling pathways leading to modulation of diverse processes.

We have previously described the effects that ouabain causes in MDCK, a cell line derived from dog kidney, widely used as epithelial model [21,22]. In concentrations in the nanomolar range, ouabain influences several physiological and structural properties of epithelia, including tight junctions, adherens junctions, apical-basolateral polarity and, more notably, gap junctional-mediated intercellular communication (GJIC) [23,24,25,26,27]. Regarding this latter property, we have shown that ouabain increases GJIC in wild MDCK cells, which are sensitive to ouabain (MDCK-S). However, this does not happen in insensitive cells (MDCK-I) in which the sequence encoding for Na^+^-K^+^-ATPase’s alpha subunit has been modified to render it insensitive to ouabain [28].

We have also described the effects that ouabain causes on the same MDCK cells at concentrations in the micromolar range. One of the most notable is that the cells detach from each other and from the substrate, although they remain viable for a certain time. Additionally, in this case we observed that there are subtypes of ouabain-resistant cells, which, unlike normal ones, do not detach. Interestingly, when both types of cells are mixed and co-cultured, the latter confer on the former the ability to resist the effect of ouabain so that they do not detach, neither from each other nor from the substrate. As we have demonstrated, this is because both types of cells establish metabolic cooperation when communicating through gap junctions [29].

Apart from describing that ouabain influences GJIC in epithelial cells, we have also demonstrated, more recently, that prostaglandin E2 (PGE2) stimulates GJIC in MDCK, although it does so through a completely different mechanism that ouabain [30].

Given these facts, in this work we focused initially on determining if under co-culturing conditions, ouabain-insensitive cells (MDCK-I) acquire the, otherwise lost, ability to increase GJIC in response to treatment with ouabain (10 nM) as sensitive cells (MDCK-S) do. As shown below, we found that in fact, MDCK-I cells acquire the ability to do GJIC, induced by a molecular component released by MDCK-S in response to treatment with ouabain. We further show that this component is PGE2.

## 2. Results

### 2.1. Cells Insensitive to Ouabain Stimulation (MDCK-I), Acquire the Ability to Establish GJIC When Co-Cultured with Sensitive (MDCK-S) Cells

First, we set out to determine whether cells insensitive to ouabain (MDCK-I) acquire the ability to communicate through gap junctions when they are co-cultured with wild, ouabain-sensitive cells (MDCK-S). For this purpose, we did dye transfer assays, to measure the gap junctional intercellular communication (GJIC), in monolayers of MDCK cells, either treated or untreated with ouabain (10 nM, 1 h). As described in detail in the Methods Section, these trials consist of randomly selecting one of the cells that make up the monolayer to inject Lucifer Yellow (LY) and count the number of cells stained because of this injection. After a certain number of trials, we calculate the average number of stained cells (${\bar{X}}_{SC}$) and consider it an estimator of GJIC. In this way we measured and compared the value of ${\bar{X}}_{SC}$ (±SE) from monolayers made from MDCK-S or MDCK-I cells as well as from monolayers made by mixing both types of cells in a proportion of 50–50%. In all three experimental conditions we made trials with and without treatment with ouabain (10 nM, 1 h).

**Figure 1:**
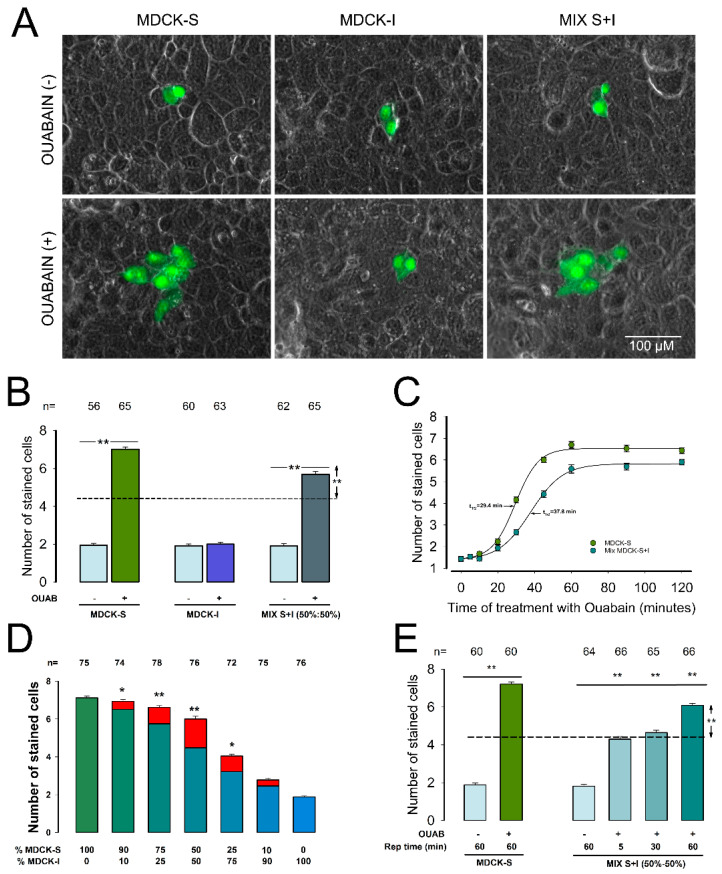
Ouabain insensitive cells (MDCK-I) are turned GJIC responsive when co-cultures with sensitive (MDCK-S) cells. (**A**) Images showing representative examples of Lucifer Yellow transfer trials. The green mark corresponds to LY dye, whereas the gray background is a phase contrast field showing the integrity of the monolayer. The images were selected to match the median of each indicated experimental condition. (**B**) Bar chat comparing the average value of the number cells stained with LY (${\bar{X}}_{SC}$) with and without treatment with ouabain (OUAB) in monolayers of MDCK-S, MDCK-I or monolayers made of mixed types. (**C**) Plot comparing the time course of the effect of ouabain in monolayers of MDCK-S and mixed monolayers. The continuous lines result from fitting data to a sigmoidal curve by non-linear regression to get (t_1/2_). (**D**) Bart chart comparing ${\bar{X}}_{SC}$ of monolayers of distinct proportions of MDCK-S and MDCK-I as indicated at the foot of bars. (*) and (**) indicate statistically significant difference, with *p* < 0.01 or *p* < 0.001 respectively, after comparing the experimental value with the theoretical, that ${\bar{X}}_{SC}$ would have in case no GJIC would occur in MDCK-I. (**E**) Bar chart comparing ${\bar{X}}_{SC}$ of MDCK-S monolayers with and without treatment with ouabain (10 nM, 1 h) and in mixed monolayers after treatment with ouabain 10 nM with media replaced at regular intervals up to 60 min. The dashed line indicates the theoretical value that ${\bar{X}}_{SC}$ would have in case of MDCK-I cells woul not communicate. (**) indicates statistically significant difference (*p* < 0.001) of the groups that separates the lines. The values above bars indicate the number of repetitions.

As illustrated in Figure 1A,B, in MDCK-S ouabain induced a significant increase in ${\bar{X}}_{SC}$ as compared to control (*p* < 0.001), from 1.9 ± 0.1 (n = 56) to 7.0 ± 0.1 (n = 65), while in MDCK-I it produced no significant difference (1.9 ± 0.1, n − 60 vs. 2.0 ± 0.1, n − 63). In monolayers produced by the mixture of 50%–−50% of sensitive and insensitive cells, ouabain also induced a significant increase in ${\bar{X}}_{SC}$ (*p* < 0.001), from 1.9 ± 0.1 (n = 62) to 5.7 ± 0.2 (n = 65), although it was significantly lower than the value obtained in monolayers consisting only of sensitive cells.

Under the 50%:50% (S/I) ratio mixing conditions, it is reasonable to assume that, if MDCK-I cells would remain without establishing GJIC, the value of ${\bar{X}}_{SC}$ would be half of that obtained from monolayers of 100% MDCK-S. Based on the values of ${\bar{X}}_{SC}$ obtained from MDCK-S monolayers, with and without ouabain (7.0 and 1.9 respectively, we calculated that ${\bar{X}}_{SC}$ in the 50:50 mixture should be 4.45 in case that MDCK-I cells remained without stablishing GJIC. Thus, the fact that the value of ${\bar{X}}_{SC}$ obtained from mixed monolayers (5.7 ± 0.2) is significantly higher (*p* < 0.01) than the theoretical value of 4.45 (*p* < 0.01), suggests that ouabain produces an additional effect, which stimulates MDCK-I cells to increase their GJIC.

To strengthen this observation, we compared the time course of the effect produced by ouabain on GJIC in mixed monolayers with that of monolayers made of only MDCK-S cells. For this purpose, we did dye transfer trials at different times of treatment with ouabain; at each given time, we calculated ${\bar{X}}_{SC}$ and plotted it against time (Figure 1C). We fitted the data to a logistic equation, using nonlinear regression, to get t_1/2_, the time at which half the effect is reached. The response obtained from mixed monolayers was noticeably slower (t_1/2_ = 37.8 min) than that obtained from monolayers of only MDCK-S (t_1/2_ = 29.4 min). This leads us to think that two different processes are involved and reinforces our assumption that, under this experimental circumstances, MDCK-I can stablish GJIC.

Given the results obtained in the assay of 50–50% (S/I) mixture, we extended the study to observe the response by varying the proportions of MDCK-S and MDCK-I cells. In each proportion ratio, we calculated the theoretical value that ${\bar{X}}_{SC}$ should have if MDCK-I cells do not have GJIC, to statistically compare the experimental value of ${\bar{X}}_{SC}$ against the corresponding theoretical value. As shown in Figure 1D, the difference is statistically significant, as the monolayers were mixed in a proportion of 90%:10% (S/I) down to 25%:75% (S/I), although the biggest difference is precisely when they are in the proportion of 50%:50% (S/I). The fact that no significant difference is observed beyond 25%:75% (S/I), led us to hypothesize that ouabain triggers the production of a signal that acts extracellularly on MDCK-I cells constituting the monolayer which, when the proportion of MDCK-S cells too low, is not concentrated enough to stimulate the MDCK-I cells. To test the hypothesis of an extracellular signal, we assayed the effect of ouabain (10 nM, 1 h) on GJIC, again in monolayers produced by mixing MDCK-S and MDCK-I in a 50:50 proportion, but this time replacing the extracellular media at fixed intervals of 5 and 30 min. If ouabain induces the production of an extracellular signal that in turn induces MDCK-I to enhance GJIC, it would be expected that replacement of the extracellular media would wash out that signal, abolishing the increased GJIC observed when the media is not replaced. Figure 1E shows the results obtained in each experimental condition performed on monolayers produced by a 50%:50% (S/I) mixture as well as the values obtained in monolayers of 100% MDCK-S. In monolayers consisting of 100% MDCK-S, the ${\bar{X}}_{SC}$ values, with and without ouabain treatment, were 1.9 ± 0.1 (n = 60) and 7.2 ± 0.1 (n = 60) respectively. In mixed monolayers, for untreated monolayers was 1.8 ± 0.1 (n = 64), while that of monolayers that were treated for 60 min with ouabain was 6.1 ± 0.1 (n = 66), which again, was significantly higher than the theoretical value (4.45) to be observed if ouabain acted only on sensitive cells. The value of ${\bar{X}}_{SC}$ obtained from monolayers that were treated with ouabain, but in which the medium was replaced every 5 min was 4.3 ± 0.1 (n = 66), while in monolayers where the medium was replaced every 30 min was 4.6 ± 0.1 (n = 65). In both cases, the values were not statistically different from the calculated theoretical value (4.45). These results, therefore, suggest that ouabain induces the release into the extracellular medium of a component that works by promoting GJIC in cells that are insensitive to the direct action of ouabain.

### 2.2. Ouabain Induces MDCK-S to Secrete a Substance That Enhances GJIC in MDCK-I

**Figure 2:**
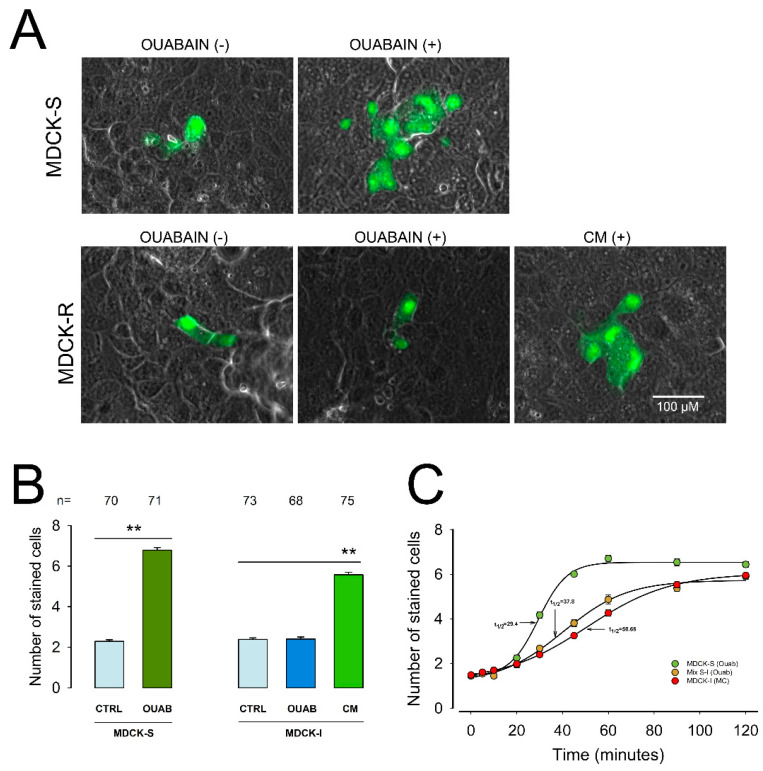
Ouabain induces in MDCK-S cells, secretion of a compound that stimulates GJIC in cells insensitive to the direct action of ouabain (MDCK-I). (**A**) Images showing representative examples of dye transfer trials. The green mark corresponds to LY, whereas the gray background is a phase contrast field showing the integrity of the monolayer. The images were selected to match the median of each indicated experimental condition. (**B**) Bar chat comparing the average value of the number cells stained with LY (${\bar{X}}_{SC}$) with and without treatment with ouabain (OUAB) in monolayers of MDCK-S and in in monolayers of MDCK-I treated with Ouabain or wit conditioned media (CM). The values above bars indicate the number of repetitions. (**) indicates statistically significant difference (*p* < 0.001) of the groups indicated by lines (**C**) Plot comparing the time course of the effect of ouabain in monolayers of MDCK-S or mixed monolayers, or MDCK-I treated with CM produced at distinct times of incubation with ouabain. The continuous lines result from fitting data to a sigmoidal curve by non-linear regression.

To further support this hypothesis, we assayed the effect of a conditioned media (CM) on the GJIC in monolayers of MDCK-I cells. CM is the culturing media collected from monolayers of MDCK-S after treatment with ouabain (10 nM) for 1 h. If ouabain induces in MDCK-S the production of an extracellular component that enhances GJIC in MDCK-I, it is expected that the CM would induce enhancement of GJIC in separate monolayers of MDCK-I cells. Figure 2A,B shows the results of an experiment in which we measure and compare ${\bar{X}}_{SC}$, on the one hand in MDCK-S cells with and without treatment with ouabain, and on the other in MDCK-I cells, treated with and without ouabain, as well as with CM. In MDCK-S cells, ouabain significantly increased ${\bar{X}}_{SC}$ (*p* < 0.001) compared to control (6.8 ± 0.1, n = 71 vs. 2.3 ± 0.1, n = 70). In MDCK-I cells, ouabain did not produce a significant effect, as ${\bar{X}}_{SC}$ in cells treated with ouabain (2.4 ± 0.1, n = 68) was not different from those untreated (2.4 ± 0.1, n = 73), however treatment with CM induced a significantly higher ${\bar{X}}_{SC}$ (5.6 ± 0.1, n = 75) as compared to control. These results, therefore, strongly support our hypothesis that ouabain induces the production of a substance that induces enhancement of GJIC.

Another approach that we assayed, to test this hypothesis, was to compare the time course of the capability of CM to induce GJIC in MDCK-I with that of GJIC by ouabain in MDCK-S. For the former condition, we varied the time (in a range of 0–120 min) that MDCK-S cells were treated with ouabain before extracting the CM, then treating MDCK-I during a fixed time of 30 min before making dye transfer trials. For the later condition we made dye transfer trials in MDCK-S at the same different times as in the former one. To compare the results, we fitted the data of both cases to a sigmoidal, four parameters equation to get the time for half the maximal effect (t_1/2_). As Figure 2C shows the time course of production of the extracellular signal in CM is slower (t_1/2_ = 50.66 min) than the response to ouabain in monolayers of only MDCK-S cells (t_1/2_ = 29.4 min) and that of cells in mixed monolayers (t_1/2_ = 37.8 min). This delay can be explained considering that some time lapse is required for ouabain to induce secretion of the extracellular component that in turn induces GJIC on MDCK-I cells.

Both results therefore strengthen the hypothesis that a component is synthesized by MDCK-S cells in response to ouabain-induced stimulus.

### 2.3. The Molecular Component Contained in CM That Causes GJIC in MDCK-I Cells Is Not a Protein

**Figure 3:**
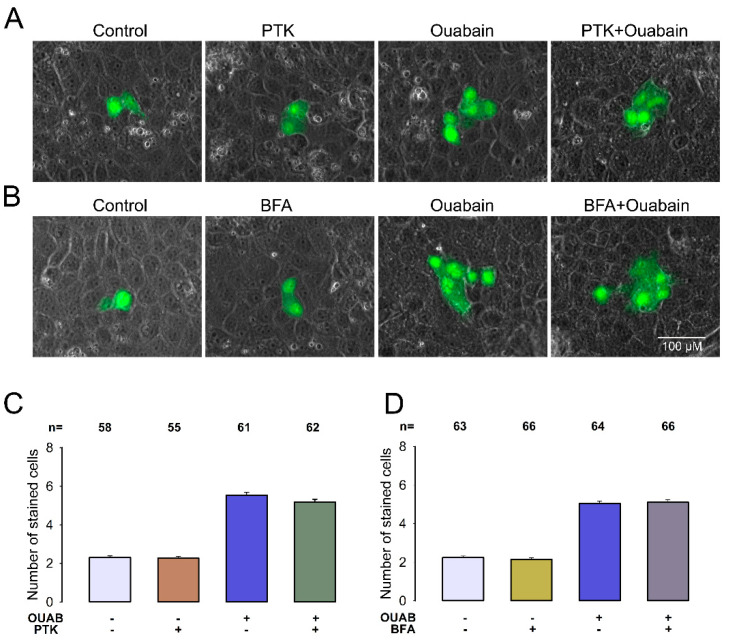
The molecular component contained in CM that causes GJIC in MDCK-I cells is not a protein. (**A**,**B**) Images showing representative examples of dye transfer trials to assay the effect of proteinase K (Pt K) and brefeldin A (BFA) respectively. The green mark corresponds to LY dye, whereas the gray background is a phase contrast field showing the integrity of the monolayer. The images were selected to match the median of each indicated experimental condition. (**C**,**D**) Bar charts comparing aNSC (±SE) in the experimental condition indicated at the bottom of each bar. (**C**) shows the effect of proteinase K and (**D**) the effect of Brefeldin A. No statistically significant difference was found between groups. The values above bars indicate the number of repetitions.

Once demonstrated that ouabain induces the production of an extracellular signal that influences GJIC, we sought to elucidate the identity of that signal. Our first assumption was that it could be a compound of a peptide nature. To probe this hypothesis, we tested the effect of proteinase K (PTK), an enzyme that is widely used to degrade protein components [31,32,33]. As shown in Figure 3A,C, CM treatment with PTK did not significantly reduce the value of ${\bar{X}}_{SC}$ compared to that obtained by dye transfer assays when treating MDCK-I cells with CM without PTK (*p* = 0.05), as the corresponding values were (5.5 ± 0.1, n = 61) and (5.2 ± 0.1, n = 62), *p* = 0.05. These results suggest therefore that the CM-contained compound is not a protein.

To strengthen this conclusion, we also tested the effect of Brefeldin A (BFA), a natural fungal metabolite which interferes with protein trafficking and secretion mediated by the Golgi apparatus and endoplasmic reticulum [34,35,36]. If the compound contained in CM is a protein, we would expect that by pretreating MDCK cells with BFA treatment with ouabain, should interfere with its secretion and therefore suppress CM’s ability to induce GJIC in MDCK-I cells. Figure 3B,D shows that the value of ${\bar{X}}_{SC}$ when MDCK-S cells were BFA pretreated with BFA prior to treatment with ouabain (5.1.1 ± 0.1, n − 66) was not significantly different (*p* − 0.85) from that obtained when they were treated only with ouabain and not BFA (5.0 ± 0.1, n − 64).

Therefore, the results obtained with PTK, as well as BFA, both indicate that the compound contained in CM is not a protein.

### 2.4. COX Inhibitors Suppress CM-Induced Enhanced GJIC in MDCK-I Cells

Since, as we have shown elsewhere, prostaglandin E2 (PGE2) induces enhancement of GJIC [30], we consider the possibility that this substance may be expressed in response to ouabain treatment and that PGE2 is therefore the substance that induces GJIC in cells insensitive to the direct action of ouabain. To test this hypothesis, we evaluated the effect of a set of compounds on the capability of CM to induce GJIC in MDCK-I cells. We tested ketorolac, indomethacin, niflumic acid, SC-560 and rofecoxib, which have been described as inhibitors of cyclooxygenase isoenzymes (COX-1 and COX-2). The procedure for evaluating the effect of those inhibitors was to incubate MDCK-S cell monolayers for 1 h with a given inhibitor before treatment with ouabain (10 nM, 1 h) to produce CM. Then treat monolayers of MDCK-I cells with the CM for 30 min before making dye transfer assays to evaluate GJICs.

**Figure 4:**
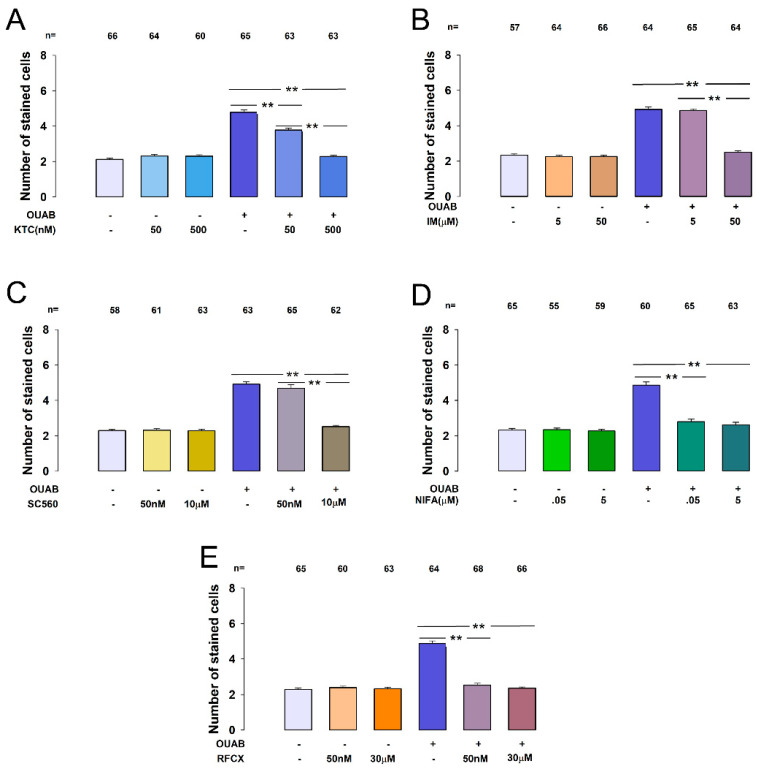
COX inhibitors suppress CM-induced enhancement of GJIC in MDCK-I cells. (**A**–**E**) are bar charts comparing ${\bar{X}}_{SC}$ (±SE) in the experimental condition indicated at the bottom of each bar. Each bar chart shows the effect of a distinct COX inhibitor (**A**) ketorolac (KTC), (**B**) indomethacin (IM), (**C**) SC-560, (**D**) niflumic acid (NIFA), (**E**) rofecoxib (RFCX). (**) indicates statistically significant difference (*p* < 0.001) of the groups that separates the lines. The values above bars indicate the number of repetitions.

We first tested the effect of ketorolac (KTC), a non-steroidal anti-inflammatory drug (NSAID) which inhibits, non-selectively, both COX-1 and COX-2 [37,38]. Since it has been reported that the IC50 of KTC is 20 nM [39], we tested the effect of 50 and 500 nM. Figure 4A shows a bar chart comparing, the values of ${\bar{X}}_{SC}$ (±SE) measured in MDCK-I cells after treatment with a CM that was obtained from MDCK-S monolayers treated with distinct experimental conditions. Those values are: control (2.1 ± 0.1, n = 66), KTC 50µM (2.3 ± 0.1, n = 64), KTC 500 µM (2.3 ± 0.1, n = 60), ouabain (4.8 ± 0.1, n = 65), ouabain + KTC 50 µM (3.7 ± 0.1, n = 63), ouabain + KTC 500 µM (2.3 ± 0.1, n = 63). These results indicate that (1) KTC itself does not exert an effect on GJIC, since the ${\bar{X}}_{SC}$ values after treatment of only KTC, were not statistically different from the control, at none of the concentrations tested (50 or 500 nM). (2) The CM obtained after treatment with ouabain produced, as described above, a statistically significant increase compared to control. (3) Pre-treatment with KTC, in both concentrations tested, significantly reduced the increase induced by treatment with only ouabain. These results support the hypothesis that PGE2 is the substance that is released into the extracellular medium after incubation of MDCK-S cells with ouabain, however, since KTC is not selective for COX1 or COX2, we tested the effect of other inhibitors, either selective for COX-1 or COX-2.

Next, we tried the effect of indomethacin (IM) a COX inhibitor which has more selectivity for COX-1 (IC50 = 1.67 µM) than for COX-2 (IC50 = 24.6 µM) [40,41]. We tested IM at 5 and 50 µM. As Figure 4B shows, the values of ${\bar{X}}_{SC}$ obtained from these assays were: control (2.3 ± 0.1, n = 57), IM 5 µM (2.2 ± 0.1, n = 64), IM 50 µM (2.3 ± 0.1, n = 66), ouabain (4.9 ± 0.1, n = 64), ouabain + IM 5 µM (4.2 ± 0.1, n = 64), ouabain + IM50 µM (2.5 ± 0.1, n = 64). The statistical analyses of this data indicates: (1) that IM does not in itself produce effect (*p* = 0.72), and (2) that pretreatment with IM 5μM does not significantly (*p* = 0.47) suppress the effect that CM has on GJIC on MDCK-I cells, but IM50μM does so (*p* < 0.001). Therefore, these results, in addition to strengthening the hypothesis that PGE2 is the substance that induces GJIC in MDCK-I cells, suggest that it is produced by COX-2 and not by COX-1.

To support this conclusion, we also tried the effect of SC-560, a highly potent, selective inhibitor of COX-1, member of the diaryl heterocycle class of cyclooxygenase (COX) inhibitors [42]. It has been described that the IC50 values for COX-1 and COX-2 are 9 nM and 6.3 µM respectively [43], therefore we tried 50 nM and 10 µM. As Figure 4C shows, the values of ${\bar{X}}_{SC}$ obtained from these assays were: control (2.3 ± 0.1, n = 58), SC50 nM (2.3 ± 0.1, n = 61), SC10 µM (2.3 ± 0.1, n = 63), ouabain (4.9 ± 0.1, n = 63), ouabain + SC50 nM (4.7 ± 0.1, n = 65), ouabain + SC10 µM (2.5 ± 0.1, n = 62). Statistical analysis of these results indicates, on the one hand, that SC-560 does not in itself have any effect on the response. However, it produced a response profile like IM: at low concentration (50 nM) did not significantly reduce the ability, induced in CM by ouabain to increase GJIC in MDCK-I cells. However, at 10 µM it did reduce GJIC significantly (*p* < 0.001).

Next, we tested niflumic acid (NIFA), which has been described as a selective inhibitor of COX-2, with IC50 values of 16 and 0.1 µM for COX-1 and -2, respectively [41,44]. We tested NIFA at 0.5 and 5 µM. As Figure 4D shows, the values of ${\bar{X}}_{SC}$ obtained from these assays were: control (2.3 ± 0.1, n = 60), NIFA0.5 µM (2.3 ± 0.1, n = 55), NIFA 5 µM (2.3 ± 0.1, n = 59), ouabain (4.9 ± 0.1, n = 60), ouabain + NIFA 0.5 µM (2.8 ± 0.1, n = 65), ouabain + NIFA 5 µM (2.6 ± 0.1, n = 63). As can be seen, NIFA significantly abolished (*p* < 0.001) the ouabain-induced effect from the concentration of 0.5 µM, reducing the response to be undistinguishable from control (*p* = 0.02), so that there is little additional effect at 5 µM. This supports the idea that COX-2 is the enzyme responsible to produce the compound that in turn induces increased GJIC in MDCK-I cells treated with CM.

Finally, we tried the effect of rofecoxib (RFCX), another member of the diaryl heterocycle class of cyclooxygenase (COX) inhibitors that selectively inhibits COX-2 over COX-1, with IC50 values of 0.018 and >15 µM, respectively [45,46]. We tested RFCX at 50 nM and 30 µM. The values of ${\bar{X}}_{SC}$ are control (2.3 ± 0.1, n = 65), RFCX 50 nM (2.4 ± 0.1, n = 60), RFCX 30 µM (2.3 ± 0.1, n = 63), ouabain (4.9 ± 0.1, n = 64), ouabain + RFCX 50 nM (2.5 ± 0.1, n = 68), ouabain + RFCX 30 µM (2.3 ± 0.1, n = 66). The statistical analyses of these data yielded similar results to that obtained from NIFA: pretreatment with RFCX50 nM abolished the ouabain-induced enhancement of GJIC in MDCK-I cells caused by CM.

Then, the results of the assays with COX inhibitors altogether, lead us to conclude that the component that is produced by MDCK-S cells in response to ouabain treatment, and which is discharged into CM is a prostaglandin, because COX inhibitors suppress CM’s ability to induce GJIC in MDCK-I cells. Moreover, we conclude that such prostaglandin is synthesized by COX-2 and not by COX-1.

### 2.5. EP2 Receptor Antagonists Abolish CM-Induced Enhancement of GJIC in MDCK-I Cells

To strengthen the hypothesis that PGE2 is the compound produced by MDCK-S cells in response to treatment with ouabain, we used another strategy, which consisted of testing the effect of compounds, known to act as antagonists of EP receptors, to which PGE2 binds [47,48,49]. If PGE2 accounts for such effect, the addition of EP antagonists to CM it is expected to abolish its capacity to enhance GJIC in MDCK-I cells.

**Figure 5:**
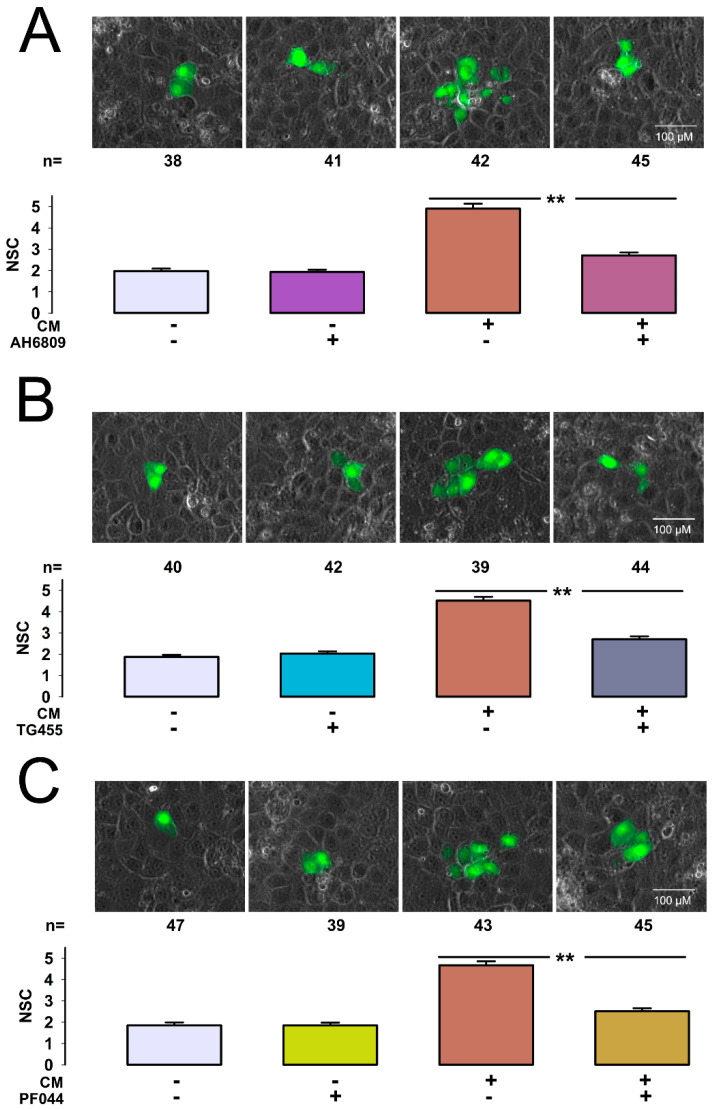
EP2 receptor antagonists abolish CM-induced enhancement of GJIC in MDCK-I cells. (**A**–**C**) sets show the effect of AH-6809 (AH), TG4155 (TG) and PF-04418948 (PF) respectively. The row of images, shown on each set, are representative images of dye transfer trials of the experimental condition shown at the bottom of the bar chart. The green mark corresponds to LY dye, whereas the gray background is a phase contrast field showing the integrity of the monolayer. The images were selected to match the median of each indicated experimental condition. The lower part of each set is a bar chart that compares the value of ${\bar{X}}_{SC}$ (±SE) of each experimental condition indicated at the bottom of each bar. (**) indicates statistically significant difference (*p* < 0.001) of the groups that separates the lines. The numbers shown above bars indicate the number of repetitions.

We first tested the effect of AH-6809 (AH), a non-selective EP antagonist. Since its EC50 has been reported to be about 1.5 µM [50], we tested 3 µM. We made dye transfer assays and measured ${\bar{X}}_{SC}$ under control conditions or after treatment with AH, or CM, or AH + CM. The results were: control (2.0 ± 0.1, n = 38), AH (2.0 ± 0.1, n = 41), CM (4.9 ± 0.1, n = 42), CM + AH (2.7 ± 0.1, n = 45). As Figure 5A shows, AH-6809 alone did not produce any effect on GJIC, as ${\bar{X}}_{SC}$ of cells treated with this compound was not statistically distinct of that of control (*p* = 0.83); however, it effectively abolished the effect induced by treatment with CM, as the value of ${\bar{X}}_{SC}$ of the group treated with CM+AH was significantly lower than that of the group treated with CM (*p* < 0.001). These results therefore support the hypothesis that PGE2 is produced by MDCK-S by treatment with ouabain, and that this compound induces GJIC in MDCK-I cells.

To further discern which EP type is involved, we examined the effect of more selective inhibitors. TG4-155 (TG) is a highly selective EP2 antagonist, with a Ki of 2.4 nM [51,52]. To try the effect of this compound we made dye transfer assays and measured ${\bar{X}}_{SC}$ under control conditions or after treatment with TG, or CM, or TG+CM. The results were: control (1.9 ± 0.1, n = 40), TG (2.0 ± 0.1, n = 42), CM (4.5 ± 0.2, n = 39), CM + TG (2.7 ± 0.1, n = 44). As Figure 5B shows, TG alone did not produce any effect on GJIC, as ${\bar{X}}_{SC}$ of cells treated with this compound was not statistically distinct of that of control (*p* = 0.26); however, it effectively abolished the effect induced by treatment with CM, as the value of ${\bar{X}}_{SC}$ of the group treated with CM+TG was significantly lower than that of the group treated with CM (*p* < 0.001). These results therefore suggest that EP2 is the receptor that mediates the CM-induced enhancement of GJIC in MDCK-I cells.

To strengthen this conclusion, we assessed the effect of PF-04418948 (PF). Another selective EP2 receptor antagonist (IC50 = 16 nM), which is over a thousand-fold less active at other EP receptors [53,54]. To try the effect of this compound we made dye transfer assays and measured ${\bar{X}}_{SC}$ under control conditions or after treatment with PF (50 nM), or CM, or PF (50 nM) + CM. The results were: control (1.8 ± 0.1, n = 47), PF (1.8 ± 0.1, n = 39), CM (4.6 ± 0.2, n = 43), CM + TG (2.5 ± 0.1, n = 45). As Figure 5C shows, PF alone did not produce any effect on GJIC, as ${\bar{X}}_{SC}$ of cells treated with this compound was not statistically distinct of that of control (*p* = 0.84); however, it effectively abolished the effect induced by treatment with CM, as the value of ${\bar{X}}_{SC}$ of the group treated with CM+PF was significantly lower than that of the group treated with CM (*p* < 0.001).

These results altogether support therefore that EP2 is the receptor that mediates the CM-induced enhancement of GJIC in MDCK-I cells.

### 2.6. Ouabain Induces a Synergistic Enhancement of GJIC

**Figure 6:**
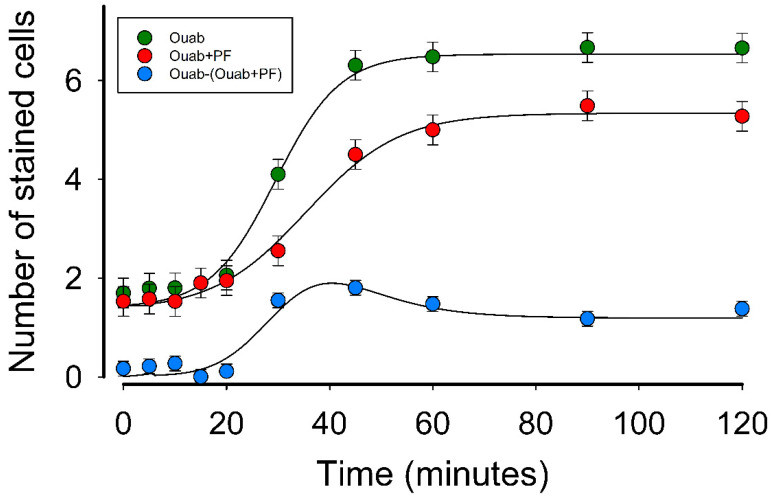
Ouabain induces a synergistic enhancement of GJIC. The plot compares the time course of ${\bar{X}}_{SC}$ in MDCK-S cells, induced by treatment with ouabain (**green circles**) or ouabain plus PF-04418948 (PF) (**red circles**) during the various periods of time as plotted. The difference from those two conditions (**blue circles**), can be attributed to PGE2´s contribution. Continuous lines show the best fit of data to a sigmoidal curve after nonlinear regression.

The results already shown suggest that in MDCK-W, ouabain induces GJIC in two ways, first by direct interaction by Na^+^-K^+^-ATPase and second by promoting paracrine secretion of PGE2, which in turn stimulates GJIC upon binding to EP2 receptor. This means that the amount of GJIC in MDCK-W cells, when treated with ouabain, results from two components. To validate this prediction and discern the time course of those components we compared the response in MDCK-S cells, on the one hand when incubated with ouabain 10 nM, with another condition in which PGE2’s contribution was ruled out by treatment with TG4-155. The results are shown in Figure 6. The fitting of data obtained in the condition of treatment with ouabain reveals a t_1/2_ = 29.4 min and a maximum ${\bar{X}}_{SC}$ of 5.1 cells. The addition of TG4-155 slowed the response (t_1/2_ = 35.4 min) and decreased ${\bar{X}}_{SC}$ to 4.0. Figure 6 also shows the time course of the difference between those two conditions, which can be considered as the component contributed by PGE2 on GJIC. As observed, it has a transient behavior, reaching a peak ${\bar{X}}_{SC}$ of 2, at 40 min of treatment, then decreasing to a steady value 1.2.

## 3. Discussion

Intercellular communication is fundamental for multicellular organisms to control all aspects of their structure and homeostasis [55]. Among the various forms of communication stands out the one that occurs between adjacent cells, either from the same tissue or from one type of tissue to another. This type of intercellular communication is carried out through gap junctions, molecular structures consisting of arrays of intercellular channels, that enable adjacent cells to communicate both electrically and metabolically [1,56,57]. Gap junctions are expressed in almost all tissues and participate in a wide variety of physiological processes, most notably those in which joint or synchronized action of the cells that make up a tissue is required, for example in the smooth or cardiac muscle or endocrine glands [58,59]. In fact, gap junctional intercellular communication (GJIC) is actively involved in virtually all aspects of the cellular life cycle, ranging from cell growth to cell death, such as cell proliferation, migration, and apoptosis [60]. They are also related to a wide variety of diseases and pathological processes, including congenital [61] or acquired disorders related, including some related to the heart [62,63,64], brain [65,66], kidney [67,68], among many others. It highlights the fact that gap junctions have been described associated in various ways with cancer [69,70,71,72], either as a cause or consequence [73]. Gap junctions and GJIC are therefore the subject of intense research in the various aspects related to these structures, including knowledge of how their expression and kinetics is modulated.

We had studied GJIC in epithelia, using MDCK, a cell line derived from dog’s kidney [21,22] as a model. Initially we described that GJIC is virtually non-existing in cells cultured as mature monolayers, nonetheless they stablish GJIC for a brief period soon after cells are harvested and re-seeded as confluent monolayers [74]. The further observation that ouabain influences the properties of epithelial cells related to cell-cell contact [75] led us to determine if ouabain influences GJIC. We had demonstrated, by dye transfer and electric coupling assays, that ouabain enhances GJIC in cells in mature monolayers. This effect occurs as a surge that reaches a maximum at the hour and then gradually decreases, to increase later, albeit more slowly. We also have described that Cx32 and Cx43 are the connexins participating in this phenomenon and that Na^+^-K^+^-ATPase plays as the receptor of ouabain that, upon binding, triggers a signaling cascade that includes cSrc and Erk1/2 [26,27]. Next, we broadened our interest to determine the effect on GJIC of digoxin and marinobufagenin, two other cardiac glycosides which, like ouabain, have been described to occur endogenously in mammals. We found that they both induce GJIC, following the same signaling pathways as ouabain does, albeit with distinct sensitivity [75]. Moreover, we have shown that ouabain induces increased GJIC in cell lines derived from different types of cancer [76]. More recently we found, interestingly, that GJIC is also modulated by prostaglandin E2, through a process that, in principle, did not appear to be related to ouabain [30].

The finding that both substances are related, was rather fortuitous. As described above, the interest to know if, under co-culturing conditions, ouabain sensitive cells would influence insensitive ones to communicate through gap junctions, led us to learn that both substances are related so that ouabain, in addition to causing increase in GJIC, induces paracrine secretion of PGE2, which in turn also acts by stimulating GJIC, so that at one point both substances act synergistically. As shown in Figure 6, a possible physiological significance of this synergism, for the cells that exhibit it, could be to respond faster and more intensely to the stimulus initiated by ouabain. Additionally, as we described, the component that is due to PGE2 induction has a transient profile. This could be because some of the components involved in the mechanism that leads to the increase in GJIC had already been synthesized and pooled, but once the pool is exhausted, de novo synthesis is required, so the response decreases and reaches a steady state.

Another interesting goal, in continuing work, will be to discern whether both pathways converge in the way that they modulate gap junctions, for this it will be necessary to carry out studies that reveal in each case the molecular identity of the connexins involved and whether the increase is due to synthesis of new components or if they rather are modulated by different post-translational modifications (PTMs) such as phosphorylation, glycosylation, proteolysis, N-acetylation, S-nitrosylation, ubiquitination, lipidation, hydroxylation, methylation and deamidation, as it has been described to occur in many instances [77,78,79,80,81].

In addition to learning more about the mechanisms and components involved, another aspect worth to investigate in the future, will be if this same synergism occurs in diverse tissues such as cardiac and smooth muscle, endocrine glands, or epithelia from diverse organs, such as kidney, lung, intestine, skin, etc. Likewise, it will be interesting to study whether this also occurs in vivo.

Although until now, there had been no description like this, neither that PGE2 modulates GJIC, nor that ouabain induces paracrine secretion of PGE2, both phenomena could be interestingly significant, especially to better understand the role that gap junctions on some physiological and pathological processes, including inflammation, hypertension, and cancer, where both ouabain and prostaglandins have been described as playing a role on their own. This, of course, could eventually lead to the proposal of new therapeutic strategies to alleviate or control those conditions.

## 4. Materials and Methods

### 4.1. Cell Culture

Starter MDCK-II cells, here referred to as MDCK-S, were obtained from the American Type Culture Collection (MDCK, CCL-34). MDCK-I, a subclone highly resistant to ouabain, was kindly provided by Dr. Louvard (Pasteur institute). For production and maintenance, cells (both MDCK-W and MDCK-R) were grown in a 5% CO_2_ atmosphere at 36.5 °C in Dulbecco’s Modified Eagle Medium (DMEM; Gibco), supplemented with penicillin-streptomycin 10,000 U/μg/mL (Cat. 15140122, Thermo Fisher Scientific, Waltham, MA, USA), and 10% fetal bovine serum (Gibco). To make dye transfer assays, cells were trypsinized and seeded at 80% confluence on glass coverslips, placed in 24-well cell culture plates, to form mature monolayers, incubated with CDMEM + 10% FBS. After 24 h the FBS in the culturing media was reduced to 1%, to avoid the possibility of interfering ouabain, and the coverslips containing monolayers were kept in this, depleted media, for 24 h. After this, the wells containing coverslips were treated with the distinct treatments described in Results before making dye transfer trials.

### 4.2. Measurement of Gap Junctional Intercellular Communication by Dye Transfer Assays

Dye transfer assays consisted of impalement and injection, with glass micropipettes, of Lucifer Yellow on individual cells from mature monolayers grown on glass coverslips. Coverslips on which cell monolayers had been grown, were placed in a translucent chamber, filled with PBS plus Ca2+ (1.8 mM) solution, at room temperature. Micropipettes were elaborated from borosilicate glass capillaries tubes (Kimax, 34500-99) on a vertical David-Kopf puller (DKI-700c, Tujunga, CA, USA). Those with a tip electrical resistance of 5–10 MOhms were backfilled with a saline solution containing 120 mM KCl, 5 mM NaCl, 1 mM MgCl_2_, and 5 mM HEPES, (pH 7.4) and Lucifer Yellow (1%). After filling up, pipettes were attached to holder device, which was mounted to a micromanipulator (PCS-750; Burleigh Instruments, NY, USA). For impalement of cells the chamber was mounted on the stage of an inverted microscope (Diaphot 300; Nikon, Tokyo, Japan) equipped with epifluorescence. Three independent trials were made. On each trial, about 30 repeats were made per coverslip. In each repeat, cells were randomly chosen from among those constituting the monolayer, then impaled and injected, one at a time, using a pneumatically driven microinjecting device (IM300; Narishige, NY, USA). After about 30 to 50 cells injected, the coverslips were rinsed with PBS and fixed by dipping into 4% paraformaldehyde, then rinsed (3×) with PBS and mounted using VECTASHIELD^®^ (H-1000; Vector Laboratories, Burlingame, CA, USA). Eight-bit images of the fluorescent cells were acquired at room temperature using a Zeiss M200 inverted microscope equipped with a Plan-NeoFluar 63 × N.A. 1.25 objective lens, an AxioCam MRm camera and software Axovision 4.8 (AXOVISION GmbH, Hanover, Germany). The captured images were imported into FIJI Is Just ImageJ software (release 2.8, NIH, Bethesda, MD, USA) to adjust the brightness and the contrast and GIMP (release 2.8.10, NIH) to compose the figures.

### 4.3. Chemicals

All chemicals and reagents were obtained from Sigma (St. Louis, MO, USA), unless otherwise noted. Lucifer Yellow was obtained from Sigma-Aldrich (67764-47-5) and dissolved in a solution containing (100 mM KCl, 5 mM NaCl, 10 mM HEPES, 1 mM CaCl_2_, pH 7.4).

TG4155 (Cat SML1217), was dissolved in DMSO at 10 mg/mL and diluted to working concentration of 5 nM. AH-6809 (Cat A1221), dissolved in DMSO at 1 mg/mL and diluted to working concentration of 700 nM. TG4155 (Cat SML1217), was dissolved in DMSO at 10 mg/mL and diluted to working concentration of 5 nM. Brefeldin A (Cat B7651), AH-6809 (Cat A1221), TG4-155 (Cat SML1217), PF-04418948 (Cat PZ0213) and SC-560 (Cat S2064) were dissolved with DMSO to stocks 20 mg/mL.

Proteinase K lyophilized powder (Cat P6556) was dissolved in water to 1 mg/mL, aliquoted and stored at −60 °C. Immediately before use the enzyme was activated by dissolving in 20 mM CaCl_2_ and kept on ice until use. CM was centrifuged for 15 min at 12,000 rpm. After addition of Proteinase K (200 µg/mL), CM was incubated at 65 oC, 1 h. Proteinase K inactivated by adding 5 mM Pefablock SC (Cat 11429868001) to CM. For treatment with Brefeldin A, MDCK-S cells in mature monolayers were incubated during 1 h with depleted media containing 50 µM Brefeldin A. Then ringed 5X before treatment with ouabain (10 nM, 1 h) to produce CM.

### 4.4. Statistical Analyses

The data collected in this work was processed and analyzed statistically using the Microsoft Office 365 Excel application and Sigmaplot 12.5. The data were generated by counting the number of cells stained with Lucifer Yellow resulting from injection of a single cell. The results shown are the product of three independent experimental trials. The number of data is indicated in the figures and in the text. The data is represented as the average value and dispersion as the standard error of the mean (SE). Statistical analysis, as indicated in the text and figures, consisted in a ONEWAY test, followed by multiple comparison tests (Holm–Bonferroni method). If the data did not meet the normality criterion (Shapiro–Wilk test) required for a parametric analysis, Kruskal–Wallis one-way ANOVA on ranks was used instead followed by nonparametric multiple comparisons (Dunn method). Simple paired comparisons were made, either with a *t*-test or Mann–Whitney rank sum test. A minimum criterion of *p* < 0.05 was considered for a statistically significant difference.

## Data Availability

Not applicable.
